# Titanium Carbide Nanofibers-Reinforced Aluminum Compacts, a New Strategy to Enhance Mechanical Properties

**DOI:** 10.3390/ma9050399

**Published:** 2016-05-20

**Authors:** Khalil Abdelrazek Khalil, El-Sayed M. Sherif, A. M. Nabawy, Hany S. Abdo, Wagih W. Marzouk, Hamad F. Alharbi

**Affiliations:** 1 Mechanical Engineering Department, College of Engineering, King Saud University, Al-Riyadh 11421, Saudi Arabia; harbihf@ksu.edu.sa; 2 Mechanical Design and Materials Department, Faculty of Energy Engineering, Aswan University, Aswan 002097, Egypt; enghany2000@yahoo.com; 3 Center of Excellence for Research in Engineering Materials (CEREM), Advanced Manufacturing Institute, King Saud University, Al-Riyadh 11421, Saudi Arabia; habdo@ksu.edu.sa; 4 Electrochemistry and Corrosion Laboratory, Department of Physical Chemistry, National Research Centre (NRC), Dokki, Cairo 12622, Egypt; 5 Production Engineering and Design Department, Faculty of Engineering, Minia Universities, Minia 61111, Egypt

**Keywords:** aluminium composites, titanium carbide, nanofibers reienforcemnts, HFIHS

## Abstract

TiC nanofibers reinforced Al matrix composites were produced by High Frequency Induction Heat Sintering (HFIHS).The titanium carbide nanofibers with an average diameter of 90 nm are first prepared by electrospinning technique and high temperature calcination process. A composite solution containing polyacrylonitrile and titanium isopropoxide is first electrospun into the nanofibers, which are subsequently stabilized and then calcined to produce the desired TiC nanofibers. The X-ray diffraction pattern and transmission electron microscopy results show that the main phase of the as-synthesized nanofibers is titanium carbide. The TiC nanofibers is then mixed with the aluminum powders and introduced into high frequency induction heat sintering (HFIHS) to produce composites of TiC nanofibers reinforced aluminum matrix. The potential application of the TiC nanofibers reinforced aluminum matrix composites was systematically investigated. 99.5% relative density and around 85 HV (833 MPa) Vickers hardness of the Al reinforced with 5 wt % TiC nanofiber has been obtained. Furthermore, the sample of Al contains 5 wt % TiC, has the highest value of compression and yield strength of about 415 and 350 MPa, respectively. The ductility of the Al/5 wt % TiC showed increasing with increasing the TiC contents.

## 1. Introduction

The past few decades have seen the extensive development of discontinuously reinforced metal matrix composites (MMCs) because of their good mechanical properties, formability, low costs and adaptable processing methods [1]. In particular, aluminum based MMCs have been widely used in the automobile, aerospace and marine sectors due to their increased stiffness, wear resistance when compared to unreinforced alloys [2,3]. Attempts to further improve the performance of MMCs have been reported by the tailoring of unique composites with microstructural control such as interpenetrating, quasi-continuous and bi-continuous composites [4,5,6,7,8]. In conventional MMCs, the most widely used reinforcements are ceramics, such as Al_2_O_3_ or SiC, in the form of fibers, flakes or particulates [6,7,8,9]. While regarding fibers at the nanoscale one of the most recently studied reinforcement for Al alloys are Carbon Nanotubes CNT [10,11,12]. Thus, aluminum based metal matrix composites reinforced by particles and fibers are successfully applied in various fields of automotive and aircraft industry. However, the interfaces between the ceramic reinforcements and the metal matrix are usually not good, which results in highly porous materials with reduced mechanical properties and increased corrosion sensitivity [13]. In order to solve this problem, metallic glasses have been recently proposed as a novel type of reinforcement in MMCs [14,15,16]. The strength of a composite material is effectively enhanced by fiber-based reinforcement. Thus, the high surface-to-volume ratio of nanofibers significantly improves the stiffness and mechanical strength of the composites compared to conventional fibers due to the increased interaction between the fibers and the matrix. Another positive aspect is that the composites are able to maintain their optical transparency related to the small cross-section of the nanofibers. In this regard, electrospinning is a technique that involves the production of continuous nanoscale to microscale sized fibers (as thin as 5 nm) from a variety of materials including polymers, composites and ceramics through the application of an electric field to a droplet of polymer solution passed through a spinneret tip. The electrospun fibers possess properties not found in conventional fibers e.g., they have a high surface to volume ratio, high aspect ratio, controlled pore size and superior mechanical performance. The superior mechanical properties associated with these fibers arise from the decrease in diameter that cannot be achieved through conventional spinning processes. Organometallic Ti compounds are selected here because they can be used as precursors of TiC [17].

On the other hand, the novel technique of high-frequency induction heat sintering (HFIHS) has been shown to be an effective sintering method that successfully consolidates ceramics and metallic powders to near their theoretical densities. The HFIHS process involves the rapid sintering of a nanostructured hard metal in a very short time with high-temperature exposure and the application of pressure. This process is advantageous because it allows for rapid densification to near the theoretical density of the associated materials and inhibits grain growth in nanostructured materials. Furthermore, the use of conventional methods of powder consolidation often results in grain growth when sintering compacts and also the presence of porosity, thus leading to compacts with lower density than the ones obtained by pressure-assisted processes. It is therefore essential to minimize grain growth through careful control of the consolidation parameters, particularly the sintering temperature, pressure and time. Furthermore, retention of the nanostructure of the final product after exposure to elevated temperatures during processing is one of the main challenges of using nanostarter powders. In this regard, high-frequency induction heat sintering (HFIHS) has been shown to be an effective sintering method that successfully consolidates ceramics and metallic powders to near their theoretical densities in a very short time. This allow rapid densification to near full density of the associated materials and inhibits grain growth in nanostructured materials. Another important reported aspect of the HFIHS process is the role of rapid heat transfer to the product via electromagnetic waves. The main objective of the present study was to help clarify and optimize the overall processing parameters of the HFIHS process, focusing on the sintering of conventional nanostructured powders and the suitability of HFIHS for the compaction of various ceramic and metallic materials. Efforts were made to reduce the grain size of the target materials to less than 100 nanometers by optimizing the overall parameters of the novel HFIHS process. The effects of processing parameters such as sintering temperatures, pressures, holding time and heating rates on the mechanical and microstructural properties were investigated systematically. In this paper, the TiC ceramic nanofibers are synthesized by electrospinning and subsequent thermal calcinations. The TiC nanofibers are then mixed with aluminum matrix. The mixture is then introduced into HFIHS using high heating rate (500 °C/min). Therefore, in this article, HFIHS method was employed to fabricate Al/TiC nanofiber nanocomposites. The effects of TiC on the microstructure and mechanical properties of Al were carefully investigated.

## 2. Experimental Details

### 2.1. Electrospinning Process

**Figure 1:**
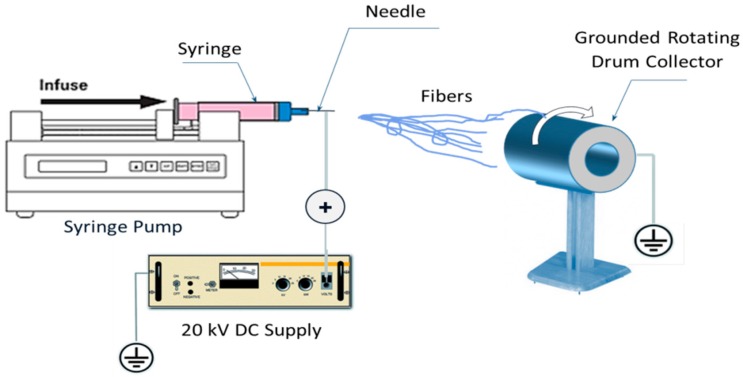
Schematic diagram of the electrospinning process.

PAN/Ti composites were prepared via sol-gel by two steps. First, 4.5 gm of titanium isopropoxide (Ti (OiPr)_4_, Aldrich), and 9 gm of acetic acid were dissolved into 30 gm of *N*,*N*-dimethylformamide (DMF). The mixture was vigorously stirred at room temperature for two hour to obtain 45 grams of a homogeneous solution. Second, polyacrylonitrile (PAN) (Mw 150,000) 7 wt % solution was dissolved in (DMF) with vigorous stirring for at least 2 h to get a transparent solution. The prepared Ti precursor solution was then dropwise added to the PAN solution with vigorous stirring to prepare a transparent PAN/Ti precursor composite solution. The sol-gel is then introduced into electrospinning machine. A schematic diagram for the used electrospinning device of polymer nanofibers in this study is shown in Figure 1.

**Figure 2:**
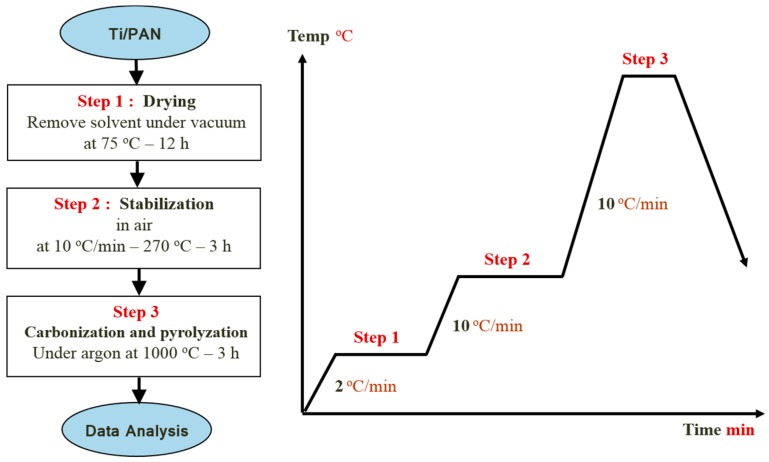
Drying and Calcination process for TiO_2_/PAN nanofibers mat.

In a typical electrospinning setup, a high-voltage source is connected to a metallic needle, which is attached to a solution reservoir (syringe) and located 15 cm from the rotating collector. The needle has a relatively small orifice of 0.2 mm that concentrates the electric charge density on a small pendant drop of solution. In the electrospinning process a high voltage (20 kV) is used to create an electrically charged jet of polymer solution with a flowrate of 0.5 mL/h. Stretching of the viscoelastic thread under the electric field combined with solvent evaporation leads to the formation of Ti/PAN nanofibers deposited on the grounded collector. Before reaching the collecting drum, the solution jet evaporates or solidifies, and then collected as an interconnected mat of small fibers. The as-spun fibers obtained from the previous step were initially dried for 12 h at 75 °C under vacuum In order to remove the polymer; binder used in making sol-gels. Samples of the dried nanofibers were placed into ceramic boats, which were then transferred to a tube furnace for calcination process. Calcination process normally takes place at temperatures below the melting point of the product materials and above the thermal decomposition temperature for decomposition and volatilization reactions) or the transition temperature (for phase transitions). The calcination process was performed by using tube furnace (CARBOLITE Type 3216CC). The tube furnace was fitted with an alumina tube with gastight fittings on each end. The samples were first stabilized thermally in the tube furnace at 270 °C in air for 3 h, and then carbonized and pyrolyzed under argon at 1000 °C for 3 h with heating rate of 10 °C/min (as shown in the schematic flowchart Figure 2).

The morphology of the nanofiber mats before and after calcination had been analyzed by JEOL JSM-7600 scanning electron microscope, JEOL Ltd., Tokyo, Japan. Transmission electron microscopy (TEM) was done by JEOL JEM 2100F operating at 200 kV, JEOL Ltd., Tokyo, Japan. Before measurement, these samples were transferred onto a Formvar-coated copper grid. Powder X-ray diffraction patterns (XRD, BRUKER, D8_Discover, Tokyo, Japan) were used to measure the crystallinity of samples, and Fourier transform infrared (FTIR) spectra were obtained using Bruker, TENSOR Series FT-IR Spectrometer, (Karlsruhe, Germany) to detect the chemical structural changes of the pyrolyzed samples.

### 2.2. Mixing Process

**Figure 3:**
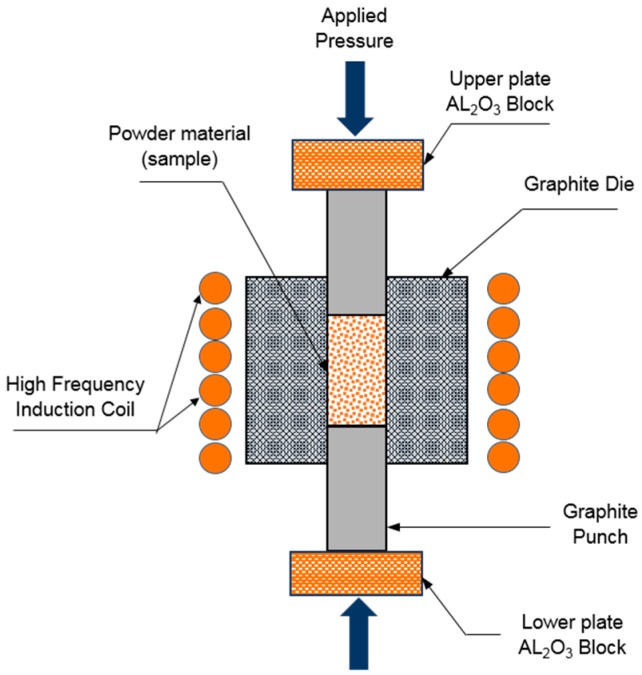
Schematic diagram of high-frequency induction heated sintering apparatus.

Pure aluminum powders (Merk, Berlin, Germany) with average size of 44 μm (325 mesh) were weighed carefully in an argon-filled glove box. All powders were milled in a Fritsch mechanical alloying machine using zirconia balls and a rotation speed of 200 rpm for 30 min with a ball-to-powder weight ratio of 5:1. Presintered electrospun TiC nanofibers with 1 wt %, 2 wt %, 3 wt %, and 5 wt % were added to the slurries and mixed with the de-agglomerated Al powder for 15 min. After mixing, the slurries were dried at 60 °C in a vacuum drying oven. The mixed composites were placed in a graphite die (outside diameter, 45 mm; inside diameter, 20 mm; height, 40 mm) and then introduced into the HFIHS machine (Figure 3).

The samples were densified by heating to a sintering temperature 580 °C and then rapidly cooled to room temperature. 50 MPa compaction pressure was applied to achieve highest density and improve the mechanical and microstructural properties. The pressure was applied in two steps. First, the pressure was maintained at a constant level during heating, and then the maximum pressure was applied during the holding time. High heating rates of about 500 °C/min were applied. Intense magnetic field is applied through the electrically conducting pressure die and, in some cases, also through the sample. Thus, the die also acts as a heating source, and the sample is heated from both the outside and inside. Temperatures was measured using a pyrometer focused on the surface of the graphite die. In this work, the uniaxial pressure is applied and an induced current (frequency of approximately 50 kHz) is then activated and maintained until densification, indicating the occurrence of sintering and the concomitant shrinkage of the sample is observed. Sample shrinkage is measured by a linear gauge that measures the vertical displacement.

### 2.3. Composites Characterization

After sintering, the samples were ground and polished for subsequent indentation and microscopy studies. The densities of the sintered samples were measured by the Archimedes principle, and the theoretical density of the composites was calculated using the rule of mixtures with a theoretical density of 2.71 g/cm^3^ for aluminum and 4.93 g/cm^3^ for titanium carbide. The microstructural observations of the polished and coated fracture surfaces of the composite were investigated using a field-emission scanning electron microscope (FE-SEM) (JEOL FESEM 7600 F) coupled with energy dispersive X-ray analyzer (EDX) analysis. The surfaces were coated with platinum to avoid charging during FE-SEM observation. Phase identification was performed on samples cut from the center of hot-pressed bodies using X-ray diffractometry (XRD, Bruker, Berlin, Germany). Hardness testing was carried out using a Vickers diamond indenter on an automated hardness tester (Buehler, Micromet5114, Microhardness tester, Akashi, Japan). Sintered specimens of Al-X wt % of TiC were tested and evaluated for their hardness. During the hardness test, a load of 2.94 N with a loading time of 10 s was applied. The specimens used in hardness testing had an average diameter of 20 mm and an average thickness of 5 mm. Three samples were tested for their hardness at three different locations. The averages of these readings were computed, reported and compared. This determination of hardness was performed at ambient temperature and followed the ASTM C 1421-99 standard. To determine the mechanical strengths of the samples under uniaxial compressive loading, the sintered Al-TiC specimens were tested in a fully automated tensile tester from Instron (Model 3385H, Instron Co., University Ave, Norwood, MA, USA) with a constant crosshead speed of 0.5 mm/min. The compressive strengths of three samples were determined, and the averages for all conditions were calculated and compared for analysis.

## 3. Results and Discussion

### 3.1. Electrospinning of the PAN/Ti Precursor Composite Nanofibers

**Figure 4:**
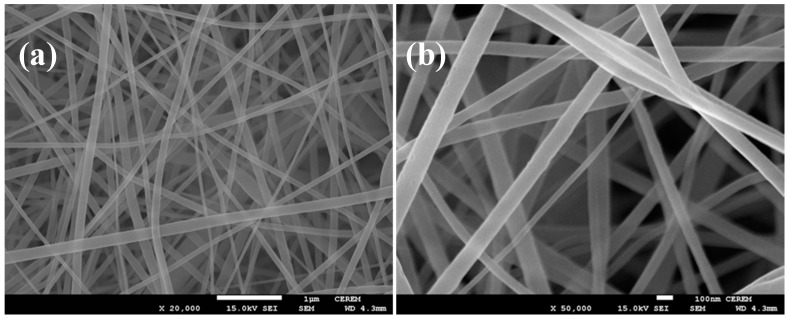
SEM images of the as-spun PAN/TTIP nanofibers before calcinations (**a**) 20 k; and (**b**) 50 k magnification.

Figure 4 shows SEM images of the PAN/TTIP nanofibers that were synthesized by electrospinning before calcination. These nanofiber composites were randomly oriented with their lengths extending to several micrometers. The morphology of the PAN/TTIP nanofibres was regular and had an average diameter of 100 nm. It was straight and the surface of the nanofibres was smooth.

**Figure 5:**
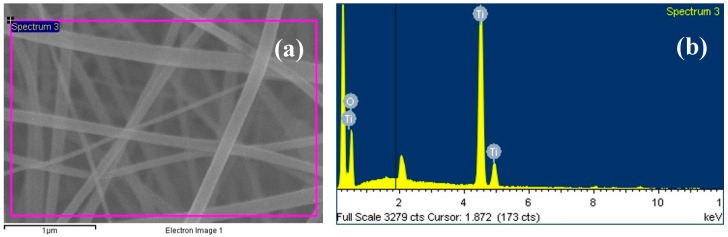
EDX results for PAN/TIIP nanofiber mat befor calcination (**a**) picked position; (**b**) elementary analysis.

In Figure 5, the energy dispersive spectroscopy (EDS) collected on the PAN/TIIP sample (whose microstructure is illustrated in Figure 4) distinctly identifies Ti as the elemental component in the fiber. EDS analysis thus provides direct evidence that Ti ions embedded in the PAN. It is indicated that titanium were well loaded without any chemical and structural modifications into PAN polymer matrix to form an organic–inorganic nanocomposite.

**Figure 6:**
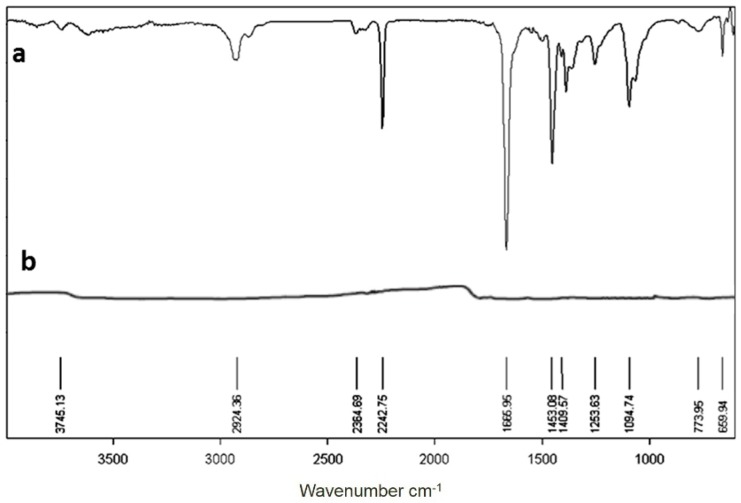
FTIR spectra of (**a**) the pure PAN; and (**b**) the calcined fiber at 1000 °C under argon atmosphere.

To produce high strength carbon fibers, oxidative stabilization of PAN prior to carbonization is the preferred route, [18,19]. The chemical structural changes of oxidative stabilization of composite nanofibers were tested using an FTIR spectrum and the result is plotted in Figure 6. For comparison, the pure PAN fibers were also analyzed and the curve is shown in Figure 6a. The most prominent structural changes are the decrease in the intensities of the 2242.75 cm^−1^, attributed to the C≡N band and the decrease of those in the regions 2930–2870, 1670–1660, 1460–1450 and 1260–1220 cm^−1^, assigned to the aliphatic CH group vibrations of different modes in CH, CH_2_ and CH_3_ [20] and the disappearance of the peaks at 1666.96 (carbonyl of AM [21], 1094 (aliphatic C–C band vibrations) [17], 1263 and 1121 cm^−1^ (the peaks of reaction products of Ti precursors), concomitant with the advent of the peaks in 1660 (due to cyclic C=O), 1608 (due to C=C), 1391 (due to C=N), and 1273 cm^−1^ (due to C–N) [22]. Compared with those results of the as-spun PAN nanofibers [20,21,22,23], there is a wide peak from 1094–773 attributed to the absorption of TiO. These changes suggest that in the thermally oxidized PAN there may be acridone, pyridine, hydronaphthiridine rings, anthrone, *etc.* [20,21,22,23,24,25,26], and they may be represented by the ladder structure. The Ti precursors may be transferred into TiO_2_. The electrospun nanofibers were further carbonized and pyrolyzed to produce the TiC–C composite nanofibers.

**Figure 7:**
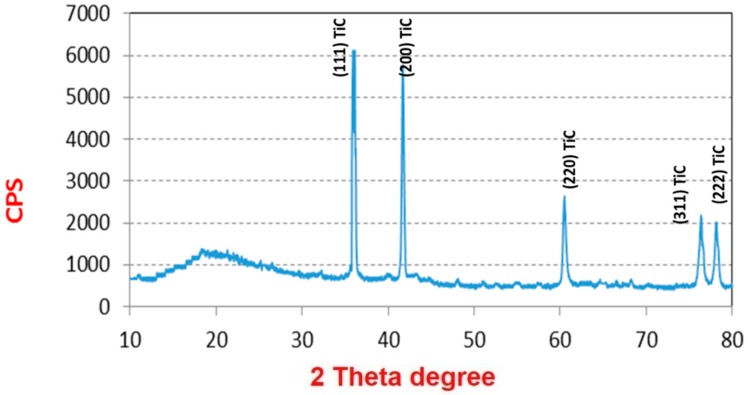
XRD patterns of the TiC calcined fiber at 1000 °C under argon atmosphere.

**Figure 8:**
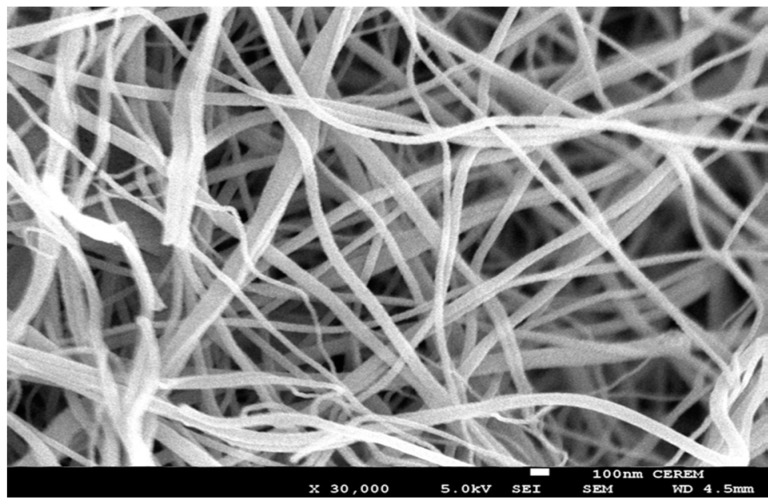
SEM images for TiC nanofiber mat after calcination at 1000 °C.

**Figure 9:**
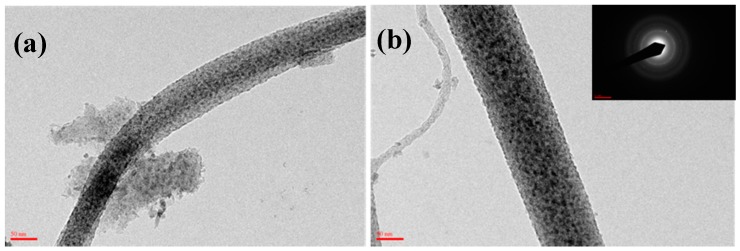
(**a**) TEM images for TiC nanofiber mat after calcination at 1000 °C; (**b**) diffraction pattern.

The XRD patterns are used to analyze the phase of the pyrolyzed fibers and the curves are shown in Figure 7. The broad peak at 20° can be attributed to carbon (002), and the other five sharp peaks at 36.9° (111), 42.6° (200), 62.0° (220), 74.48° (311) and 78.18° (222) can be well assigned to TiC [15]. The FTIR spectrum combining with the XRD curve can well confirm that no TiO exists in the fibrous matrices. This result demonstrated that carbonization of the as-spun fibers in air can facilitate carbon and titanium carbide to crystallize. Figure 7 and Figure 8 shows the SEM and TEM images (see Figure 9) of the high temperature pyrolyzed composite nanofibers. These pictures show that the TiC–C nanofibers have slightly smaller diameter, 90 nm, than the as-spun nanofibers.

The reduction of the fibrous diameter is due to the fibrous shrinkage and the reduction in composition. The inset in Figure 8 is a selected-area electron diffraction pattern taken from the square area of the nanofiber image, demonstrating further that the pyrolyzed nanofibers are the TiC-containing products. This result is consistent with the above XRD result.

### 3.2. Consolidation of Al-TiC Composites

**Figure 10:**
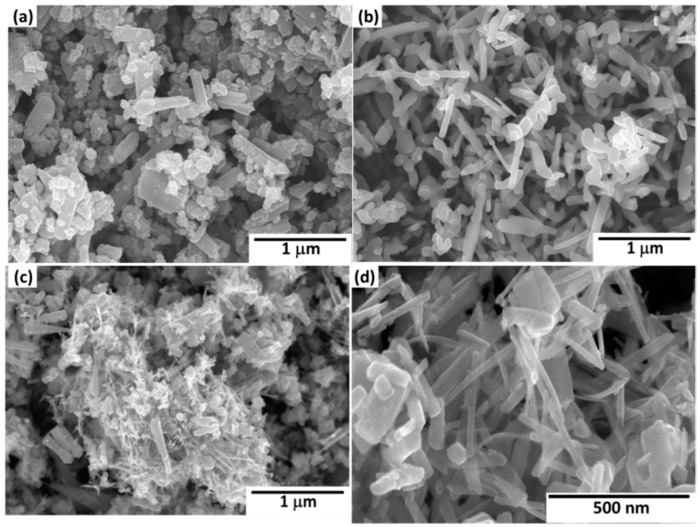
SEM micrographs of the Al/TiC nanofibers after mixing: (**a**) 1 wt % TiC; (**b**) 2 wt % TiC; (**c**) 3 wt % TiC; (**d**) 5 wt % TiC with high magnifications.

Figure 10a–d shows morphologies of the Al-TiC electrospun nanofiber after de-agglomeration and mixing. From this observation, it is confirmed that the TiC electrospun fibers were broken into elongated particles with aspect ratio (length to the diameter of the nanofibers) nearly 5 to 15. In Figure 10d, the enlarged image, the nanofibers appeared with dense microstructure due to the high calcination temperature. Thus, milling results in uniform distribution of Al and TiC electrospun nanofibers.

**Figure 11:**
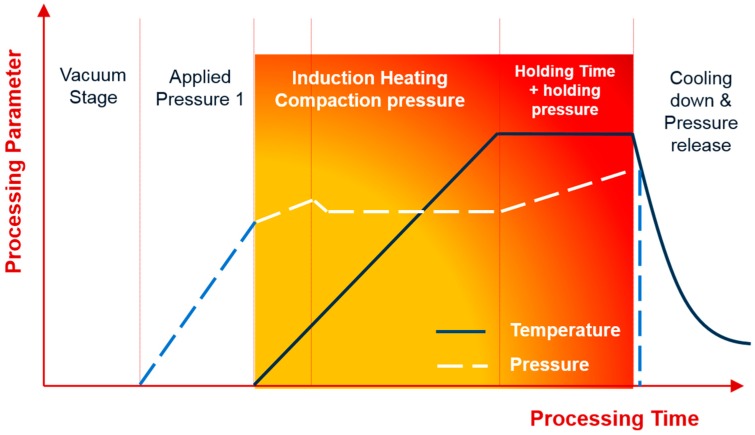
The 5 major stages of sintering process in the HFIHS machine.

The basic configuration of a HFIHS unit (ELTek Co., Gyeonggi-do, Korea) is reported elsewhere [27,28,29]. The five major stages of the HFIHS and densification processes are shown in Figure 11.

**Figure 12:**
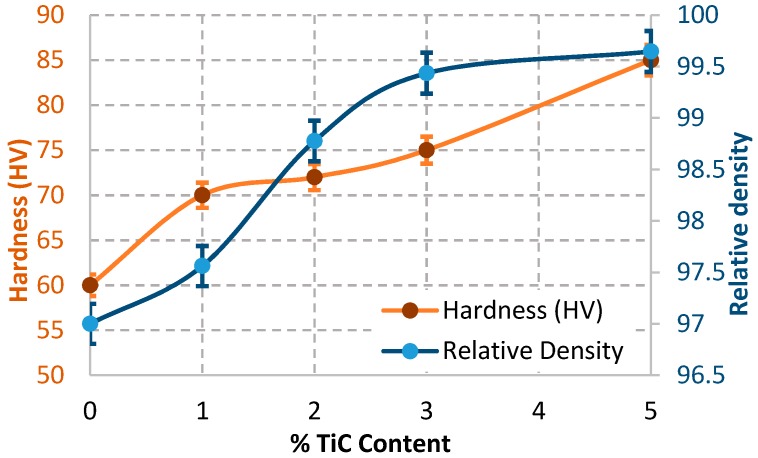
Relative density and Vickers hardness of the Al/TiC nanocomposites with different TiC contents.

Figure 12 shows the relative density and Vickers hardness of the Al/TiC nanocomposites depending on the amount of TiC additives. The relative density of the Al increased with increasing TiC nanofiber additives, reached as high as 99.5% with Al-5 wt % TiC contents. Furthermore, the Vickers hardness has greatly increased with increasing the amount of TiC contents. The hardness of Al reinforced with TiC reached maximum, around 85 HV (833 MPa) in the samples contains higher percentage of TiC content, as shown in Figure 12.

Due to mixing process, the TiC electrospun fibers were broken into small parts with aspect ratio nearly 5 to 15. Some fragmented TiC nanoparticles also exist. Notably, the relative density of the Al/TiC electrospun nanofiber almost did not vary with the increasing the TiC contents more than 3 wt %, being on the same level of approximately 99.5% as shown in Figure 12.

**Figure 13:**
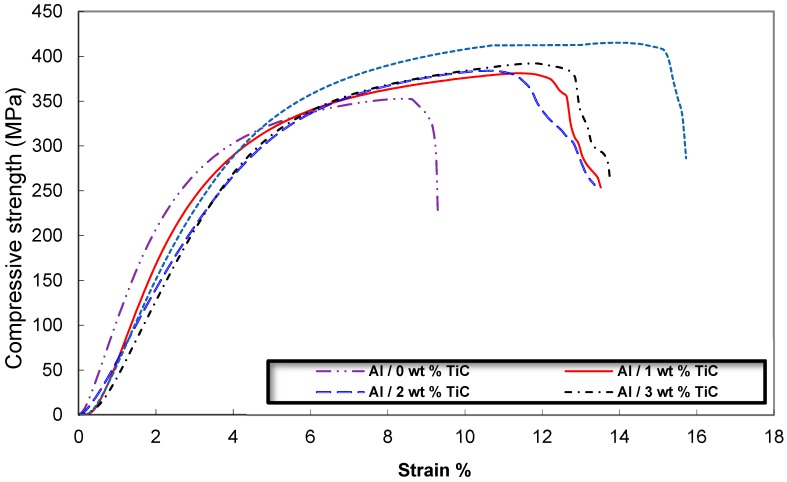
Typical stress-strain diagram of the Al/TiC nanocomposites with different TiC contents.

**Figure 14:**
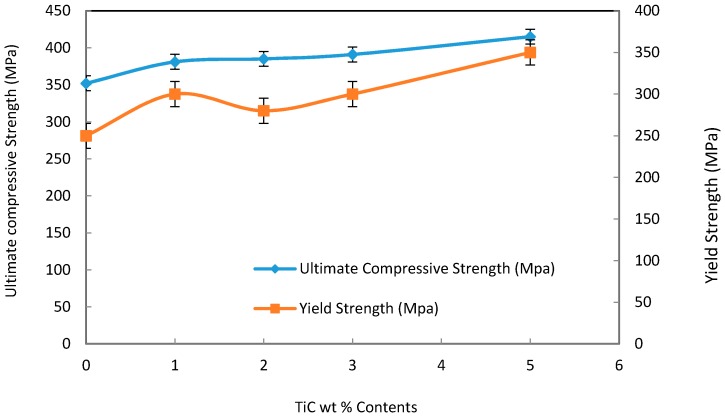
The dependence of yield and compressive strength of Al on the TiC nanofiber contents.

**Figure 15:**
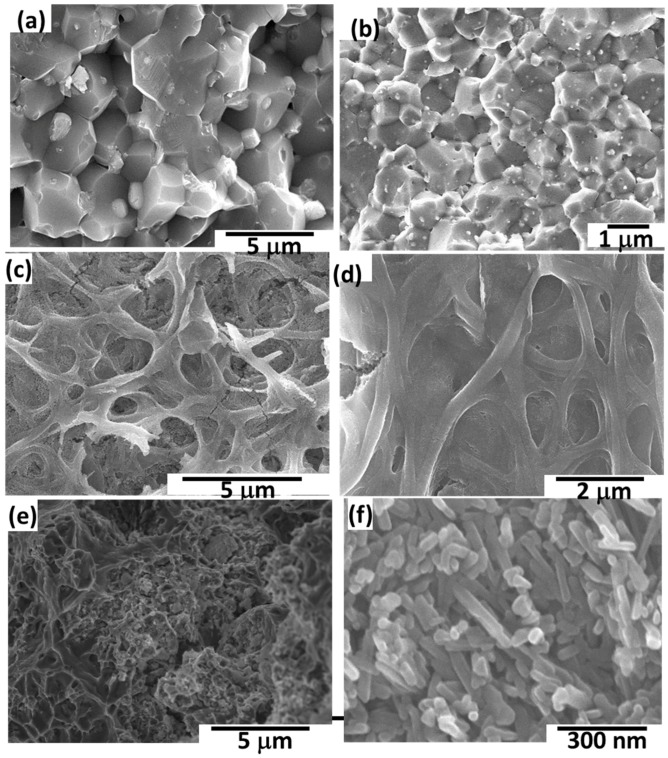
SEM micrographs of fracture surfaces of Al/TiC nanofibers composites: (**a**) Pure Al; (**b**) 1 wt % TiC; (**c**) 2 wt % TiC; (**d**) 3 wt % TiC; (**e**) 4 wt % TiC; (**f**) 5 wt % TiC.

Figure 13 shows the typical stress-strain curve of the Al/TiC electrospun nanofiber composites depending on the TiC contents. The average result of three compressive tests carried out under the same conditions for each specimen was obtained. Figure 14 shows the dependence of compressive and yield strength (average value for three compression test specimens) on the TiC nanofiber contents. It is clear that, the sample of Al contains 5 wt % TiC, has the highest value of t compression and yield strength about 415 and 350 MPa, respectively. Unexpected results that the ductility of the Al/TiC showed increasing with increasing the TiC contents. This may be attributed to the pull out resistance of the reinforcement nanofibers during fracture. The pull out mechanisms were clearly explained from the SEM micrographs of the fracture surfaces and the indentation crack propagation paths for the composites. As shown in Figure 15, the TiC nanofibers are homogeneously dispersed within the Al matrix and almost distribute at the grain boundary of the Al-TiC composites. There are a large number of residual holes left by the pulling out of the TiC nanofibers.

SEM micrographs of the fracture surfaces of Al containing TiC electrospun nanofibers contents are shown in Figure 15a–c. The addition of electrospun nanofibers led to improve the densification process and eliminate the pores. As shown in Figure 15b,c, the orientation and distribution of TiC nanofiber in the composites is homogeneous. The exposed fractured ends of TiC nanofiber could be clearly observed. They suggest that the bonding between Al and TiC nanofiber was strong so that the TiC nanofiber could easily immerse in the matrix and enhanced the strength of the Al. It can be also observed that there are no gaps around TiC nanofiber, and that the matrices of composites were dense enough under the present experimental conditions. The TiC nanofibers residing at the crack front might also effectively cause crack bridging due to stresses associated with the particulate inclusions. Nanofibers could be seen embedded in the fine Al matrix.

**Figure 16:**
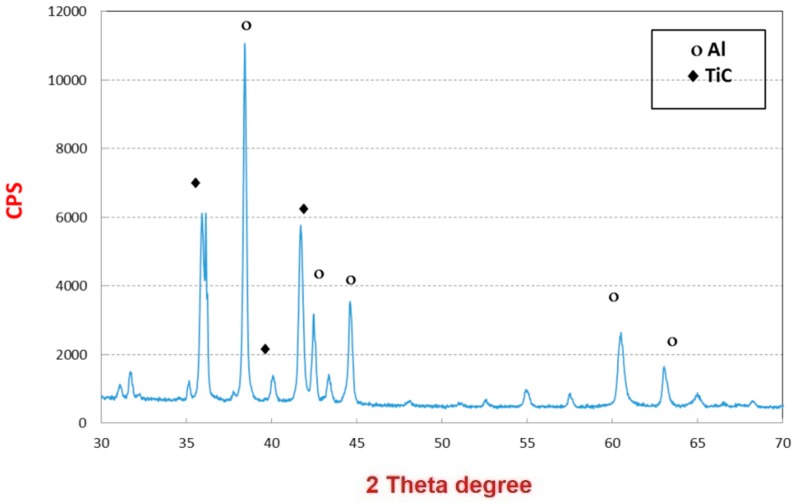
XRD patterns of the Al/TiC nanofibers after HFIHS.

XRD patterns of the pure Al/TiC nanofibers after HFIHS pressing in the same conditions are shown in Figure 16. Compared with these profiles, Al phase was the main constituent phase and some peaks corresponding to TiC are observed in the composite sample. There is no new crystalline phase formed during the sintering by HFIHS indicating that no chemical reactions between Al and the TiC happened.

## 4. Conclusions

High Frequency Induction Heat Sintering (HFIHS) have been used to produce nanocomposites of TiC nanofibers reinforced Al matrix composites. The titanium carbide nanofibers were first synthesized by sol-gel followed by electrospinning technique and high temperature calcination process. The average diameter of the calcined nanofibers was 90 nm. The formation of the as-synthesized titanium carbide nanofibers was confirmed by X-ray diffraction pattern and transmission electron microscopy. The TiC nanofibers is then mixed with the aluminum powders and introduced into (HFIHS) to produce composites of TiC nanofibers reinforced aluminum matrix. Compaction of the mixture was at a temperature of 580 °C under the pressure of 50 MPa. A high dense Al-TiC of 99.5% relative density and around 85 HV (833 MPa) hardness has been obtained in the Al reinforced with 5 wt % TiC nanofiber. With respect to mechanical properties, the samples of Al contains 5 wt % TiC, have as high as 415 and 350 MPa, respectively value of compressive and yield strength.

## References

[1] Clyne T.W., Withers P.J. (1993). An Introduction to Metal Matrix Composites.

[2] Miracle D.B. (2005). Metal matrix composites—From science to technological significance. Compos. Sci. Technol..

[3] Chawla N., Chawla K. (2006). Metal Matrix Composites.

[4] Huang L.J., Geng L., Peng H.X., Zhang J. (2011). Room temperature tensile fracture characteristics of *in situ* TiBw/Ti6Al4V composites with a quasi-continuous network architecture. Scr. Mater..

[5] Huang L.J., Wang S., Dong Y.S., Zhang Y.Z., Pan F., Geng L., Peng H.X. (2012). Tailoring a novel network reinforcement architecture exploiting superior tensile properties of *in situ* TiBw/Ti composites. Mater. Sci. Eng. A.

[6] Peng H.X., Fan Z., Evans J.R.G. (2001). Bi-continuous metal matrix composites. Mater. Sci. Eng. A.

[7] Binner J., Chang H., Higginson R. (2009). Processing of ceramic–metal interpenetrating composites. J. Eur. Ceram. Soc..

[8] Hansen N. (1969). Strengthening of aluminium by a three-dimensional network of aluminium-oxide particles. Acta Metall..

[9] Zhang D.L., Koch C.C., Scattergood R.O. (2009). The role of new particle surfaces in synthesiszing bulk nanostructured metallic materials by powder metallurgy. Mater. Sci. Eng. A.

[10] Carvalho O., Miranda G., Soares D., Silva F.S. (2013). CNT-reinforced aluminum composites: Processing and mechanical properties. Ciênc. Tecnol. Mater..

[11] Zhu X., Zhao Y.G., Wu M., Wang H.Y., Jiang Q.C. (2016). Effect of initial aluminum alloy particle size on the damage of carbon nanotubes during ball milling. Materials.

[12] Carvalho O., Miranda G., Soares D., Silva F.S. (2016). Carbon nanotube dispersion in aluminum matrix composites—Quantification and influence on strength. Mech. Adv. Mater. Struct..

[13] Montazeri A., Javadpour J., Khavandi A., Tcharkhtchi A., Mohajeri A. (2010). Mechanical properties of multi-walled carbon nanotube/epoxy composites. Mater. Des..

[14] Bhaduri A., Gopinathan V., Ramakrishnan P., Miodownik A.P. (1996). Processing and properties of SiC particulate reinforced Al–6.2Zn–2.5Mg–1.7Cu alloy (7010) matrix composites prepared by mechanical alloying. Mater. Sci. Eng. A.

[15] Sun Z.Q., Zhang D., Li G.B. (2005). Evaluation of dry sliding wear behavior of silicon particles reinforced aluminum matrix composites. Mater. Des..

[16] Slipenyuk A., Kuprin V., Milman Y., Goncharuk V., Eckert J. (2006). Properties of P/M processed particle reinforced metal matrix composites specified by reinforcement concentration and matrix-to-reinforcement particle size ratio. Acta Mater..

[17] Dudina D.V., Georgarakis K., Li Y., Aljerf M., LeMoulec A., Yavari A.R., Inoue A. (2009). A magnesium alloy matrix composite reinforced with metallic glass. Compos. Sci. Technol..

[18] Lee M.H., Kim J.H., Park J.S., Kim J.C., Kim W.T., Kim D.H. (2004). Fabrication of Ni–Nb–Ta metallic glass reinforced Al-based alloy matrix composites by infiltration casting process. Scr. Mater..

[19] Scudino S., Surreddi K.B., Sager S., Sakaliyska M., Kim J.S., Löser W., Eckert J. (2008). Production and mechanical properties of metallic glass-reinforced Al-based metal matrix composites. J. Mater. Sci..

[20] Jiang Z., Rhine W.E. (1991). Preparation of TiN and TiC from a Polymer Precursor. Chem. Mater..

[21] Severini F., Formaro L., Pegoraro M., Posca L. (2002). Chemical modification of carbon fiber surfaces. Carbon.

[22] Paris O., Loidl D., Peterlik H. (2002). Texture of PAN- and pitch-based carbon fibers. Carbon.

[23] Liang C.Y., Pearson F.G., Marchessault R. (1961). The tertiary CH and CD vibrational frequencies in polyacrylonitrile and α-deutero polyacrylonitrile. Spectrochim. Acta.

[24] Zhang W., Liu J., Wu G. (2003). Evolution of structure and properties of PAN precursors during their conversion to *carbon* fibers. Carbon.

[25] Laffont L., Monthioux M., Serin V., Mathur R.B., Guimon C., Guimon M.F. (2004). An EELS study of the structural and chemical transformation of PAN polymer to solid carbon. Carbon.

[26] Chen J.C., Harrison I.R. (2002). Modification of polyacrylonitrile (PAN) carbon fibre precursor via post-spinning plasticization and stretching in dimethyl formamide (DMF). Carbon.

[27] Khalil A., Almajid A.A. (2012). Effect of high-frequency induction heat sintering conditions on the microstructure and mechanical properties of nanostructured magnesium/hydroxyapatite nanocomposites. Mater. Des..

[28] Kim S.W., Khalil K.A. (2006). High frequency induction heating sintering of mechanically alloyed alumina-yttria stabilized zirconia nano-bioceramics. J. Am. Ceram. Soc..

[29] Khalil K.A., Kim S.W. (2006). Effect of processing parameters on the mechanical and microstructural behavior of ultra-fine Al_2_O_3_- (ZrO_2_+8%Mol Y_2_O_3_) bioceramic, densified by high frequency induction heat sintering. Int. J. Appl. Ceram. Tec..

